# Antibiotic use during pregnancy: how bad is it?

**DOI:** 10.1186/s12916-016-0636-0

**Published:** 2016-06-17

**Authors:** Amir A. Kuperman, Omry Koren

**Affiliations:** Blood Coagulation Service and Pediatric Hematology Clinic, Galilee Medical Center, Nahariya, Israel; Faculty of Medicine in the Galilee, Bar-Ilan University, Henrietta Szold St. 8, POB 1589 Safed, Israel

**Keywords:** Microbiome, Gut, Fetus, Placenta, Amniotic fluid, Pregnancy, Antibiotics, Immune system

## Abstract

**Background:**

Our microbial companions (the “microbiota”) are extremely important for the preservation of human health. Although changes in bacterial communities (dysbiosis) are commonly associated with disease, such changes have also been described in healthy pregnancies, where the microbiome plays an essential role in maternal and child health outcomes, including normal immune and metabolic function in later life. Nevertheless, this new understanding of the importance of the microbiome has not yet influenced contemporary clinical practice regarding antibiotic use during pregnancy.

**Discussion:**

Antibiotic treatment during pregnancy is widespread in Western countries, and accounts for 80 % of prescribed medications in pregnancy. However, antibiotic treatment, while at times lifesaving, can also have detrimental consequences. A single course of antibiotics perturbs bacterial communities, with evidence that the microbial ecosystem does not return completely to baseline following treatment. Antibiotics in pregnancy should be used only when indicated, choosing those with the narrowest range possible.

**Summary:**

Bacteria are essential for normal human development and, while antibiotic treatment during pregnancy has an important role in controlling and preventing infections, it may have undesired effects regarding the maternal and fetoplacental microbiomes. We expect that microbiota manipulation in pregnancy, through the use of probiotics and fecal microbiota transplantation, will be the subject of increasing clinical interest.

## Background

The human body is home to a variety of microorganisms, termed the microbiota, consisting of up to 100 trillion bacterial cells, most of which reside in the gut. Large sequencing efforts, such as the Human Microbiome Project, have characterized the microbiota of the major sites (gut, mouth, skin, airways, and vagina) of the human body in healthy individuals and demonstrated that different body sites harbor diverse populations of microbes [1]. Our microbial companions are extremely important for the preservation of human health, and a growing number of studies describe how changes in these bacterial communities are linked to disease states such as obesity [2], diabetes [3], atherosclerosis [4], and autoimmune disorders [5], among others. These shifts in community structure are termed dysbiosis. Although dysbiosis is commonly associated with disease, it has also been described in healthy pregnancies in which there is a bidirectional relationship between pregnancy and the microbiome, whereby pregnancy affects the composition of the microbiome and the microbiome plays a role in maternal and child health outcomes [4]. These shifts in microbial composition occur in several sites in the body of the pregnant woman.

## Maternal gut and vaginal microbiome changes during normal pregnancy and pregnancy complications

Pregnancy is characterized by profound hormonal, immunological, and metabolic changes aimed at supporting the growth of the fetoplacental unit [6]. Interestingly, a healthy pregnancy also induces dramatic changes in the maternal gut microbiota over the course of gestation, with a great expansion of diversity between individuals, an overall increase in Proteobacteria and Actinobacteria, and reduced diversity within each microbiome. Similarly, the vaginal microbiome in pregnancy is different from that of non-pregnant women, with lesser diversity and richness, and with a dominance of *Lactobacillus* species, Clostridiales, Bacteroidales, and Actinomycetales [7]. When transferred to germ-free mice, third trimester microbiota induced greater adiposity and low-grade inflammation compared to first trimester microbiota [4], and it was recently suggested that alteration of the gut microbiota during pregnancy may also induce complications of pregnancy such as excessive maternal weight gain [8].

Maternal pregnancy complications significantly influence the bacterial composition and diversity of the stool microbiota of premature infants, with these changes persisting during the first year after birth [9]. Antibiotic usage during pregnancy undoubtedly affects the bacterial environment of the mother and of the fetus.

## Bacteria of the fetoplacental unit – more fact than fiction?

**Fig. 1:**
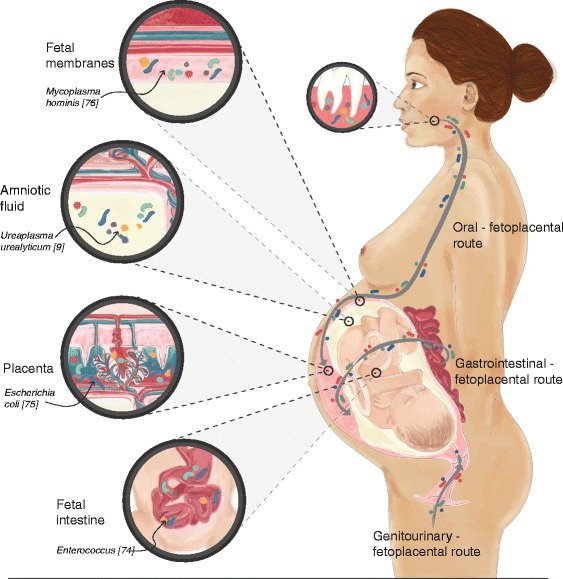
The fetoplacental microbiome of healthy pregnancy and its origins. Bacteria and their genes have been isolated from human placentas, amniotic fluid, fetal membranes, and fetal gastrointestinal tract in healthy, normal pregnancies. These bacteria have three main routes of entry: the oro-fetoplacental route, the gastrointestinal-fetoplacental route, and the genitourinary-fetoplacental route. Examples of specific bacteria are indicated

**Fig. 2:**
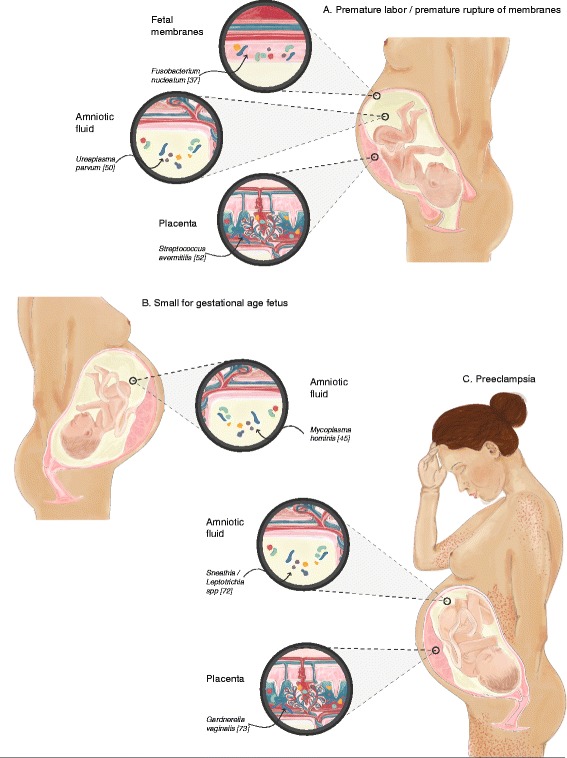
The fetoplacental microbiome in various pregnancy complications involving the placenta. Examples of specific disease-associated species are shown. **a** Bacteria are found in the placenta (*Streptococcus avermitilis*), fetal membranes (*Fusobacterium nucleatum*), and amniotic fluid (*Ureaplasma parvum*) in cases of premature labor and premature rupture of membranes. **b** Bacteria are present in amniotic fluid (*Mycoplasma hominis*) in small-for-gestational-age (intrauterine growth restriction) fetuses. **c** Bacteria are present in the placenta (*Gardnerella vaginalis*) and amniotic fluid (*Sneathia/Leptotrichia spp*) in cases of preeclampsia. A pregnant woman is illustrated, exhibiting headache, edema, and petechia

We are rapidly progressing towards understanding the role of bacteria in novel target organs traditionally considered “sterile”, including the central nervous system [10], blood [11], the lower airways [12], the sub-epidermis [13], and the fetoplacental unit [14]. ‘*The fetus lies in a sterile environment*’ declared the French pediatrician Henry Tissier of the Pasteur Institute in 1900 [15]; since then, the placenta was traditionally believed to form a sterile barrier between the colonized maternal urogenital tract and the fetus. However, recent studies have challenged this assumption. The first demonstration of bacteria in the placenta in the modern scientific literature was published in 1982 by Kovalovszki et al. [16]. Subsequent publications have described the placental microbiome, using culture-dependent and -independent methods, and suggested its relative similarity to the oral microbiome, with bacteria such as *Prevotella tannerae* and *Neisseria* being present in the placenta [14] (Fig. 1). Specific components of the placental microbiome have been linked to particular pregnancy complications (Table 1, Fig. 2). Several mechanisms of amniotic fluid colonization have been proposed, including the translocation of vaginal bacteria [17] via the bloodstream or from the oral cavity [18]. Other possible routes include hematogenous spread or bacterial migration from the lower gastrointestinal tract to the lower genitourinary tract (Fig. 1).

**Table 1:** Summary of studies providing evidence for bacteria in the human fetoplacental unit in both complicated and uncomplicated pregnancies (✓marks published evidence)

Pregnancy state	Method	Placenta	Amniotic fluid	Fetal membranes	Reference
Normal pregnancy	Microscopy	✓	✓	✓	[26, 37–40]
	Bacterial cultures	✓	✓	✓	[16, 37, 39, 41–50]
	Culture independent methods	✓	✓	✓	[14, 26, 37, 38, 41, 43, 45, 50–62]
Premature rupture of membranes and preterm labor	Microscopy	✓		✓	[41, 61]
	Bacterial cultures	✓	✓		[16, 20, 39, 44, 50, 52, 57, 63–69]
	Culture independent methods	✓	✓	✓	[14, 16, 19, 20, 39, 42, 46, 50, 52, 53, 61, 70]
Intrauterine growth restriction	Bacterial cultures		✓		[71]
	Culture independent methods		✓		[45, 72]
Pre-eclampsia	Bacterial cultures	✓	✓		[46, 72, 73]
	Culture independent methods	✓	✓		[49, 51, 58, 62, 72, 73]

The fetal membranes consist of the amniotic membrane, which contains the amniotic fluid, and the chorion, which is in proximity with the decidua parietalis. It has been shown that certain bacteria may be present in fetal membranes without leading to an inflammatory response [16]; Galask et al. [19] demonstrated that bacteria can traverse intact fetal membranes. Other bacteria resident in the fetal membranes are thought to play a role in amniotic inflammation, and thus in the initiation of labor [20].

Some of the studies described above demonstrated very low levels of bacterial DNA in the fetoplacental components. As detection methods become more sensitive, even more rigorous precautions must be taken against contamination when screening for bacterial colonization [21]. Nevertheless, despite the possibility of sample contamination, the physiological and beneficial presence of bacteria in the placenta is gaining widespread acceptance.

## Antibiotic use in pregnancy - an opinion

Perhaps the most clinically relevant aspect of the pregnancy microbiome is antibiotic treatment during pregnancy. Antibiotics account for 80 % of all prescribed medication in pregnancy [22], yet surprisingly, few published human studies have carefully evaluated the direct effects of antibiotics during pregnancy on either the maternal or fetal microbiome, or evaluated long-term sequelae of such antibiotic use. Thus, there may be a reason for caution in prescribing antibiotics during pregnancy.

In pregnant NOD mice, antibiotic treatment caused alteration of gut microbiota and immunological changes in the intestine of the offspring [23]. In pregnant women, it was demonstrated that antibiotic administration during pregnancy leads to alterations in the vaginal microbiome prior to birth, with long-term effects on the early microbial colonization of the newborn [24] and an association with childhood obesity [25].

There are several components to this issue. Antibiotic treatment of infectious diseases is one of the greatest advances of modern medicine. Accordingly, antibiotics are widely prescribed during pregnancy as the most important modality for treating and preventing infections. It is estimated that one in five pregnant women in Europe is prescribed at least one antibiotic during pregnancy; in the United States, the rate is double [26]. Nevertheless, prescription of antibiotics should be carefully considered on an individual basis, weighing its benefits versus drawbacks for both the fetus and the mother. It has been shown that administration of certain antibiotics is linked to a significantly higher rate of neonatal necrotizing enterocolitis, although antibiotic treatment is also associated with a reduced rate of lung complications and major cerebral abnormalities, relative to non-antibiotic treated controls [27]. A more recent study published in 2008 demonstrated that the prescription of antibiotics for women in spontaneous preterm labor with intact membranes was associated with an increased risk of cerebral palsy and functional impairment among their children at 7 years of age [28].

As discussed above, the healthy microbiome is important for maintaining a normal pregnancy and, therefore, it has been suggested that we may be using too many antibiotics during pregnancy [29]. A large systematic review concluded that antibiotics during the second and third trimester do not reduce adverse pregnancy outcomes and morbidity [30]. In addition, even a short course of antibiotics perturbs bacterial communities in human hosts [30]. In one study, it was shown that, within 30 days following cessation of antibiotic treatment, fecal microbiota reached an average similarity of 88 % to baseline, with the level rising to 89 % within 60 days [31]; however, the microbiota did not completely return to baseline over the timescale studied. Thus, antibiotics cause an immediate perturbation of the ecosystem, followed by incomplete recovery of the gut microbiome. The response to a given antibiotic is individualized, and may be influenced by prior exposure to the same drug. Accordingly, even a short course of antibiotics may sometimes have a long lasting residual effect on the microbiome, with possible metabolic or immune consequences.

The use of antibiotics during pregnancy has also been associated with increased risk of asthma in early childhood [32–34], increased risk of childhood epilepsy, and increased risk of childhood obesity [25]. Of course, the argument could be made that the primary maternal infection was the cause for the increased risk of these conditions, rather than the treatment itself. Nevertheless, we suggest that antibiotics in pregnancy may affect the bacterial ecosystem of the mother as well as that of the fetus, and therefore that their use should be carefully considered based on what is known, and what remains unknown, regarding their effects.

Recent studies have demonstrated that priming of the immune system and microbiota-driven immune changes begin in utero and are not – as traditionally believed – induced postnatally by the newborn’s microbiota [35]. These new insights suggest that the maternal microbiota during pregnancy actually drives early postnatal innate immune development [36]. It is becoming clearer that the maternal microbiota, in concert with maternal antibodies, are important in preparing the fetus for host-microbial symbiosis later in life. The mechanisms of this phenomenon are now being explored and involve microbial molecular transfer (without any live bacteria). In addition, maternal antibodies have a dual effect, promoting pathogen neutralization whilst simultaneously enhancing microbial molecular transfer. Gomez de Aguero et al. [36] recently showed that pups of mothers transiently colonized during pregnancy have a greater capacity to avoid inflammation in response to bacterial molecules and penetration of intestinal microbes. Thus, the maternal microbiota plays a role in shaping the postnatal immune system and interferences with maternal microbiota during pregnancy may hinder the natural process of prenatal immune priming.

We believe that the issue of antibiotics during pregnancy is one of the greatest challenges of human microbiome research and certainly deserves increased focus in the form of observational and interventional studies to unravel the role of these drugs in human development.

## Summary and future directions

One may argue for the importance of antibiotics during pregnancy to prevent or treat bacterial infections. Indeed, antibiotics have an important role in improving and promoting health in pregnant women. Nevertheless, as with other therapeutic modalities, overuse may be counter-productive. The realization that antibiotic use in pregnancy may interfere with the delicate balance between the pregnant woman’s microbiota, which is important for normal fetal development, may gradually decrease the enormous, and possibly excessive, utilization of these drugs during pregnancy. In case of proven maternal infection, narrow spectrum antibiotics should be preferred due to their less extensive effects on the microbiome, taking into account the association of prenatal antibiotics with increased risk of childhood asthma, epilepsy, and obesity. Future studies should focus on maternal microbiome manipulations such as personalized probiotics and fecal microbiota transplantation, and their effect on pregnancy outcome, as well as on the use of specific microbial profiling for diagnosis of pregnancy complications and prediction of long-term outcomes of newborn and maternal health.
